# Changes in Functional Capacity and Body Composition After a Multimodal Prehabilitation Program in Patients with Cancer undergoing Abdominal Surgery

**DOI:** 10.1245/s10434-026-19173-4

**Published:** 2026-02-23

**Authors:** Luuk D. Drager, Baukje van den Heuvel, Dieuwke Strijker, Cornelis J. H. M. van Laarhoven, Laurien M. Buffart, Sjors Verlaan

**Affiliations:** 1 Department of Surgery, Radboud University Medical Center, Nijmegen, The Netherlands; 2 Department of Medical Biosciences, Radboud University Medical Center, Nijmegen, The Netherlands; 3 Department of Nutrition and Dietetics, Faculty of Health, Sport and Physical Activity, Amsterdam University of Applied Sciences, Amsterdam, The Netherlands

**Keywords:** Prehabilitation, Exercise, Nutrition, Oncology, Surgery

## Abstract

**Background:**

Preoperative functional capacity and body composition are associated with postoperative outcomes in patients undergoing abdominal surgery for cancer. Long-term effects of multimodal prehabilitation and exercise dose–response associations remain unclear. This study aimed to investigate changes in functional capacity and body composition during and after prehabilitation and evaluate exercise dose–response associations.

**Methods:**

Data from 816 patients who participated in the monocenter stepped wedge F4S PREHAB trial (March 2021–April 2024) and underwent elective abdominal surgery for gastrointestinal, gynecological, or urological cancers were included. Functional capacity measures included estimated peak oxygen uptake, maximum short exercise capacity, muscle strength (leg press, handgrip), and five-times sit-to-stand test. Body composition measures included weight, fat-free mass, percentage body fat, and phase angle. Assessments occurred at baseline, after prehabilitation, and 3 months postoperatively. Changes over time and dose–response associations were evaluated with linear mixed models.

**Results:**

Maximum proportions of missing data ranged from 12.9% at preintervention and 24.9% at postintervention to 62.4% after surgery. Significant improvements over time were observed in estimated peak oxygen uptake (*β*=0.6 mL/kg/min, 95% confidence interval [CI] 0.4–0.8), maximum short exercise capacity (*β*=17.5 Watt, 95% CI 13.8–21.2), leg press (*β*=18.9 kg, 95% CI 15.9–21.9), handgrip (*β*=1.1 kg, 95% CI 0.6–1.6), sit-to-stand time (*β*=0.9 seconds, 95% CI -1.1 to -0.6), body weight (*β*=1.1 kg, 95% CI 0.7–1.4), and fat-free mass (*β*=0.5 kg), 95% CI 0.1–0.8). Some exercise dose–response associations were found. Functional capacity measures returned to or declined below baseline value. Sensitivity analyses with complete data at follow-up yielded comparable findings with attenuated associations. Associations varied by tumor type, neoadjuvant therapy, malnutrition risk, and training compliance.

**Conclusions:**

Functional capacity and body composition improved after prehabilitation, although gains were generally not sustained postoperatively. Associations may vary between patient subgroups. Future research should focus on targeted approaches and integration with improved postoperative rehabilitation.

*Trial registration*: The F4S PREHAB trial is registered in the International Clinical Trials Registry Platform, NL8699. Registered on 05-06-2020.

**Supplementary Information:**

The online version contains supplementary material available at 10.1245/s10434-026-19173-4.

## Background

A significant proportion of patients undergoing major abdominal surgery experience postoperative complications.^1^ These complications are associated with increased mortality, prolonged hospital stays, and greater healthcare costs.^2,3^ Previous studies showed that patients with cancer often experience a loss of skeletal muscle mass and decline in functional capacity during the preoperative period.^4,5^ Preoperative functional capacity and body composition were associated with postoperative outcomes.^6–8^ Hence, preventing functional decline and loss of muscle mass during the preoperative period may be important to optimize postoperative outcomes.^9^

Multimodal prehabilitation, often including but not limited to physical exercise, nutritional support, psychological support, smoking and alcohol cessation, and correction of anemia, has emerged as a promising approach to enhance preoperative functional capacity in patients scheduled for surgery.^10,11^ An increasing number of studies are examining the effects of multimodal prehabilitation in patients undergoing colorectal cancer surgery, with some demonstrating beneficial effects on postoperative complications and enhanced postoperative recovery,^12,13^ whereas others report no significant reductions in postoperative complications.^14,15^

Furthermore, knowledge of the course of functional capacity and body composition throughout both the preoperative and the postoperative periods remains limited,^16^ particularly in patients undergoing abdominal surgery for cancer types other than colorectal.^13,17–19^ Moreover, the exercise dose–response relationship of prehabilitation on changes in functional and body composition outcomes remain largely unknown. These knowledge gaps limit the ability to optimize and personalize prehabilitation programs for individual patients.

Therefore, in this study, we aim to examine changes in functional capacity and body composition after a multimodal prehabilitation program during the preoperative and postoperative periods in a large cohort of patients with cancer undergoing major abdominal surgery. Additionally, we aim to evaluate exercise dose–response associations for these outcomes.

## Methods

### Participants and Study Design

In the present study, we used data from the F4S PREHAB trial, which is a monocenter stepped wedge trial designed to evaluate the hospital-wide effects of a multimodal prehabilitation program in patients undergoing various high-impact surgical procedures.^20^ From March 1, 2021, multimodal prehabilitation was introduced sequentially, one clinical pathway per month, with the order determined by logistical feasibility. Patients were informed about the study procedures by a member of their clinical care team and subsequently referred for an intake and baseline assessment. Written informed consent was obtained prior to study participation. The local Medical Ethics Committee (METC Oost-Nederland; NL73777.091.20) approved the F4S PREHAB trial.

For the present sub-study, we included patients who were allocated to the intervention group and underwent elective abdominal surgery for cancer between March 1, 2021, and April 30, 2024, in the Radboudumc (Nijmegen, the Netherlands). Abdominal surgery for cancer included gastrointestinal cancers (colon cancer, rectal cancer, liver cancer or colorectal liver metastases, peritoneal metastases, retroperitoneal sarcomas, esophageal cancer, and pancreatic cancer), gynecological cancers (endometrial and ovarian cancer), and urological cancers (bladder and renal cancer).

This sub-study represents a more detailed evaluation of functional capacity and body composition within the trial cohort. These outcomes were prespecified in the original trial protocol. The present analyses focus on the sub-cohort of patients undergoing abdominal surgery using a predefined analyses plan developed shortly after trial initiation. Patients were excluded from participating in the F4S PREHAB trial if they had contraindications for high-intensity exercise defined by recommendations of the American College of Sports Medicine (e.g. unstable cardiovascular, metabolic, or renal disease)^21^ or for protein supplementation (i.e., chronic kidney disease stage  ≥ 4), an American Society of Anesthesiologists (ASA) score  ≥ 4, or inability to read or understand the Dutch language.

### Prehabilitation Program

The F4S PREHAB intervention contained a tailored multimodal prehabilitation program for a minimum of 3 weeks. The program comprised a physical exercise program, nutritional support, psychological support, a smoking cessation program, alcohol cessation advice, and correction of anemia.

The exercise program consisted of three 60 minute sessions per week supervised by a local physiotherapist close to a patient’s home and included both high-intensity interval training (HIIT) on a cycle ergometer and resistance exercise training. The HIIT component started with a 2 minute warm-up followed by alternating intervals of 4 minutes at high intensity (at 90% of the maximum short exercise capacity [MSEC], estimated by the Steep Ramp Test [SRT]) and 3 minutes at light intensity (at 30% of MSEC). The MSEC was estimated by the SRT at baseline.^22^ The SRT started after a 3-minute warming-up process without resistance. During the test, the workload increased by 25W every 10 seconds, starting at 25W. The test ended when the cycling pedal frequency fell below 60 rpm. The MSEC was calculated as the workload of the last completed stage plus 2.5W for each second in the current stage.^23^ Absolute workload was increased when participants reported a Borg rating of perceived exertion score  < 15 or demonstrated a heart rate  < 85% of their estimated maximum during the high-intensity intervals. The highest recorded heart rate observed during the baseline SRT was used as the participant’s estimated maximal heart rate for guidance during training sessions. The workload was reduced when participants reported a Borg rating of perceived exertion score of  ≥ 18. Resistance training targeted major muscle groups through six exercises: leg press, chest press, abdominal crunch, low row, lat pulldown, and step up. Patients performed two sets of 10 repetitions of machine-based exercises. The initial training load was set at 65% of the estimated one-repetition maximum (1RM), with a progressive increase of 5% per week. To ensure patient safety,^24^ 1RM was derived from an indirect 1RM protocol.^25^ If patients were receiving neoadjuvant treatment, functional tests at baseline were performed  ≥ 14 days after the last administration to minimize acute treatment effects. On non-supervised days, patients were advised to engage in 60 minutes of moderate-intensity aerobic exercise.

Nutritional support was provided by a registered in-hospital dietitian, who offered individualized dietary guidance to optimize energy, micronutrient intake, and a daily protein intake of at least 1.5 g/kg bodyweight. Each patient received a baseline consultation with a dietitian that included screening for malnutrition (e.g., weight loss, body mass index [BMI], and dietary intake), with additional sessions provided as needed. To support nutritional goals, patients were supplied with high-quality whey protein shakes (containing 30 g of whey protein and 20 µg vitamin D) and daily multivitamin supplements covering 50% of the recommended daily intake. Patients were advised to consume the protein shakes daily and directly after each supervised training session.

Psychological support was offered when indicated based on Hospital Anxiety and Depression Scale scores  ≥ 15. This cut-off has been shown to provide high sensitivity and specificity for detecting syndromal depression in patients with cancer.^26^ Referrals were made to clinical psychologists, who provided individualized counseling sessions focusing on anxiety reduction, stress management, and coping strategies tailored to the upcoming surgical procedure (e.g., relaxation techniques, cognitive restructuring, and practical preparation guidance). Finally, patients who were active smokers were provided access to a comprehensive smoking cessation program (SineFuma), and all patients received instructions to abstain from alcohol consumption during the preoperative period.

### Outcome Measures

Main outcome measures of this study were changes in functional capacity and body composition over time. Measurements were conducted at baseline prior to prehabilitation, following prehabilitation prior to surgery, and 3 months after surgery.^20^ The post-surgical measurement was added to the study protocol via an amendment 18 months after the study began and 222 (27.2%) patients had been enrolled.

Functional capacity was evaluated through aerobic fitness, muscle strength, and function. Aerobic fitness was evaluated using the MSEC (Watts) and estimated peakVO_2_ (mL/kg/min) based on the SRT on a cycle ergometer (Lode, Groningen, the Netherlands).^22^ Leg muscle strength was examined using an indirect 1RM (kg) on a leg-press machine.^25^ Handgrip strength (HGS; kg) was measured three times on each hand using a hydraulic handgrip dynamometer (Jamar, Sammons Preston, Bolingbrook, USA), with the highest value recorded. Lower body functioning was assessed with the five-times sit-to-stand (FTSST), for which patients were instructed to rise from a chair five consecutive times as quickly as possible without using their hands.^27^ The time required to complete the task (in seconds) was recorded. Body composition parameters, including phase angle (degrees), fat-free mass (FFM; kg), and percentage body fat (%) were determined using bioelectrical impedance analysis (Bodystat 1500). Anthropometric measurements and BIA assessments were conducted first, followed by the physical performance tests.

### Covariables

Baseline characteristics and clinical data such as age, sex, smoking status, ASA score, and type of neoadjuvant treatment were collected from electronic medical records. BMI (kg/m^2^) was calculated using measured body height and weight. Malnutrition risk was assessed using the Patient-Generated Subjective Global Assessment Short Form (PG-SGA SF), with scores categorized into low risk (0–3) and medium to high risk ( ≥ 4).^28^ Attendance at exercise sessions and adverse events during training sessions were recorded by local physiotherapists in an exercise log, and compliance with other treatment modalities was assessed through patient-reported outcomes during the postintervention measurement.

### Statistical Analyses

Descriptive statistics were used to describe the characteristics of the study population. Changes in functional capacity and body composition were analyzed using linear mixed model analyses, with separate models for each outcome. In all models, we included age, sex, smoking status (no vs. yes), ASA score (I–II vs. III), tumor location (gastrointestinal, gynecological, urological), neoadjuvant treatment (no vs. yes), and malnutrition risk based on PG-SGA SF scores (0–3 vs.  ≥ 4) as fixed effects, and patient as a random effect. Regression coefficients (expressed in the unit of measurement) and corresponding 95% confidence intervals (CIs) were reported and interpreted to assess potential clinically relevant changes over time. To evaluate the robustness of the findings considering missing postoperative data, we performed a sensitivity analysis in a subgroup of patients with complete data at both baseline (prior to surgery) and the 3-month postoperative follow-up.

To assess whether these changes over time were modified by tumor location, neoadjuvant therapy status, malnutrition risk, adjuvant therapy status, or exercise session compliance (analyzed both categorically based on median split [0–6 vs.  > 6 sessions] and continuously), we added interaction terms of these variables with timepoint into the model. In case of statistically significant interaction, changes over time were analyzed separately within the subgroups.

A *p*-value ≤ 0.05 was considered statistically significant. All statistical analyses were performed using IBM SPSS, version 29.

## Results

**Fig. 1:**
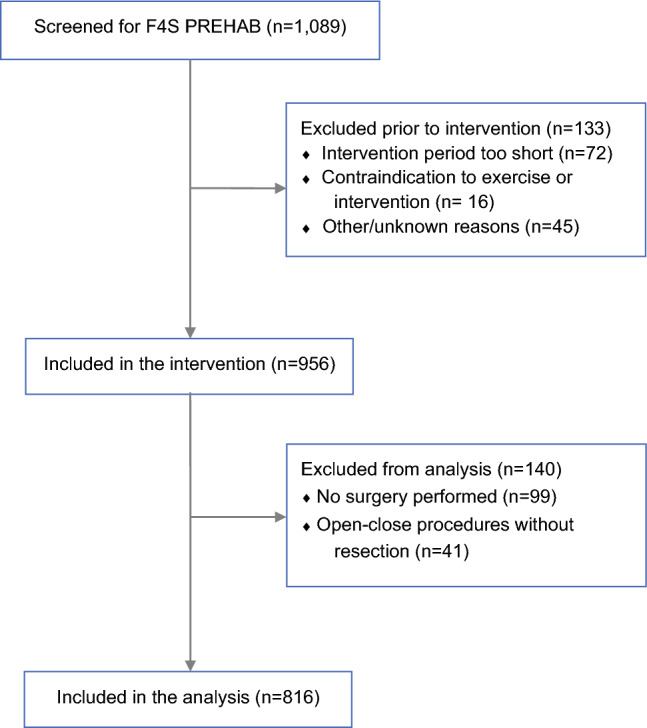
Participant flow chart

In total, 1089 patients were screened for prehabilitation, 133 of whom were excluded before participating in the intervention (Fig. 1). An additional 140 patients were excluded because they did not have a surgical resection, either because of complete response or metastatic disease (e.g., open-close procedures) (Fig. 1). Data from 816 patients with cancer were included in the analyses; the mean age was 64.2 ± 11.9 years, and 55.1% were male (Table 1). The mean BMI was 27.0 ± 4.8 kg/m^2^, and 12.5% were current smokers. Most patients (65.4%) had an ASA score of I or II. Tumor locations were distributed across gastrointestinal (71.8%), gynecological (14.0%), and urological (14.2%) malignancies. A total of 35.1% of patients received neoadjuvant treatment, and 23.7% received adjuvant treatment.

**Table 1:** Baseline characteristics

Characteristic	Prehabilitation cohort (*n* = 816)
Age, years	64.2 ± 11.9
Male sex	450 (55.1)
BMI, kg/m^2^	27.0 ± 4.8
Smoking status	
Current	102 (12.5)
Former	400 (49.1)
Never	313 (38.4)
ASA score	
I–II	534 (65.5)
III	282 (34.5)
Tumor location	
*Gastrointestinal*	586 (71.8)
Colon	34 (4.2)
Esophagus	136 (16.7)
Liver	124 (15.2)
Pancreas	107 (13.1)
Peritoneum	58 (7.1)
Rectum	85 (10.4)
Retroperitoneum	42 (5.1)
*Gynecological*	114 (14.0)
Endometrium	63 (7.7)
Ovarium	51 (6.3)
*Urological*	116 (13.2)
Bladder	60 (7.4)
Kidney	56 (6.9)
Neoadjuvant treatment	
*No*	529 (64.9)
*Yes*	286 (35.1)
Chemotherapy	117 (14.3)
Radiotherapy	16 (2.0)
Chemoradiotherapy	153 (18.8)
Adjuvant treatment	
*No*	623 (76.3)
*Yes*	193 (23.7)
Chemotherapy	109 (13.4)
Radiotherapy	20 (2.5)
Chemoradiotherapy	13 (1.6)
Immunotherapy	51 (6.3)
Exercise sessions	6 (3–9)
>6 exercise sessions	395 (48.4)

Data are presented as mean ± standard deviation, n (%), or median (interquartile range)

ASA, American Society of Anesthesiologists; BMI, body mass index

The median intervention duration was 18 days (interquartile range [IQR]10–27.5). During prehabilitation, patients followed a median of six exercise sessions (IQR 3–9) with a local physiotherapist (Table 1). One patient experienced a vasovagal reaction during HIIT and was subsequently evaluated in the emergency department. In total, 674 patients (82.6%) used protein and vitamin supplementation on a daily basis during the intervention period. A total of 108 patients (13.2%) had one or more sessions with a medical psychologist. Almost half of the 102 active smokers participated in the smoking cessation program (47.1%), and 24 patients (23.5%) successfully quit during the intervention period.

Missing data regarding functional capacity and body composition measurements varied per test and timepoint: preintervention (1.3–12.9%), postintervention (13.6–24.9%), post-surgical follow-up (54.9–62.4%). Missing values at the postintervention time point were primarily due to logistical reasons, most commonly the inability to perform tests on the morning of surgery when patients were admitted and operated on the same day. The largest proportion of missing data occurred at the postoperative measurement, as this assessment was incorporated into the study at a later stage.

### Changes in Functional Capacity Over Time, Modifying Factors, and Dose–Response Associations

Following the prehabilitation period, estimated peakVO_2_ increased significantly compared with baseline (*β*=0.6 mL/kg/min, 95% CI 0.4–0.8) (Table 2). Increases were significantly larger among patients who had undergone neoadjuvant therapy (*p*_interaction_ = 0.002) and those who completed more than six exercise sessions (*p*_interaction_= 0.036) (Table 2). A significant exercise dose–response association was observed (per exercise session: *β*=0.05 mL/kg/min, 95% CI 0.02–0.09). At post-surgical follow-up, estimated peakVO_2_ had returned to baseline levels.

**Table 2:** Descriptives of functional capacity parameters at all timepoints and changes over time with interaction terms

Functional capacity parameters	Preintervention	Postintervention	Post-surgical follow-up (3 months)	Difference between baseline and postintervention, β (95% CI)^a^	Difference between baseline and post-surgical follow-up, β (95% CI)^a^
*Functional capacity*					
Estimated peakVO_2_, mL/kg/min	(n=733)	(n=624)	(n=330)		
*Total population*	20.7 (20.2–21.2)	21.3 (20.8–21.8)	20.7 (20.2–21.3)	**0.6 (0.4–0.8)**	0.0 (− 0.2–0.3)
*Neoadjuvant therapy*^*b*^					
No	20.9 (20.3–21.5)	21.3 (20.7–21.9)	20.8 (20.2–21.4)	**0.4 (0.2–0.6)**	− 0.1 (− 0.4–0.2)
Yes (p_interaction_=0.002)	20.3 (19.3–21.2)	21.2 (20.2–22.1)	20.5 (19.5–21.5)	**0.9 (0.6–1.2)**	0.2 (− 0.2–0.7)
*Exercise sessions*					
0–6	20.6 (19.9–21.2)	21.0 (20.3–21.6)	20.6 (19.9–21.3)	**0.4 (0.1–0.7)**	0.1 (− 0.3–0.4)
>6(p_interaction_=0.036)	20.9 (20.2–21.7)	21.7 (20.9–22.5)	20.9 (20.1–21.7)	**0.7 (0.5–1.0)**	0.0 (− 0.4–0.3)
*Change per exercise session*				**0.05 (0.02–0.09)**	0.01 (− 0.04–0.06)
MSEC in Watt	(n=733)	(n=624)	(n=330)		
*Total population*	182.2 (174.9;189.5)	199.7 (192.3–207.1)	176.0 (168.3–183.8)	**17.5 (13.8–21.2)**	**− 6.2 (− 10.9 to − 1.4)**
*Tumor location*^*b*^					
Gastrointestinal	182.3 (174.7–189.8)	201.2 (193.5–208.8)	173.1 (164.8–181.5)	**18.9 (14.4–23.4)**	**− 9.1 (− 15.3 to − 3.0)**
Urological	177.3 (156.5–198.1)	185.7 (164.7–206.7)	175.6 (154.3–197.0)	8.4 (− 1.1–18.0)	− 1.6 (− 11.9–8.6)
Gynecological (p_interaction_=0.020)	149.5 (131.6–167.3)	168.0 (149.9–186.1)	147.0 (128.4–165.6)	**18.6 (10.3–26.8)**	− 2.5 (− 12.2–7.3)
*Neoadjuvant therapy*^*b*^					
No	191.0 (182.4–199.6)	203.4 (194.6–212.1)	184.1 (175.0–193.2)	**12.3 (7.8–16.9)**	**− 6.9 (− 12.5 to − 1.3)**
Yes					
(p_interaction_≤0.001)	168.2 (154.0–182.3)	194.4 (180.1–208.7)	162.5 (147.5–177.5)	**26.2 (19.9–32.6)**	− 5.7 (− 14.3–2.9)
*Exercise sessions*					
0–6	172.4 (162.2–182.6)	184.8 (174.4–195.3)	166.6 (155.7–177.6)	**12.4 (7.2–17.7)**	− 5.8 (− 12.4–0.9)
>6 (p_interaction_=0.004)	190.1 (179.5–200.6)	211.6 (201.0–222.2)	183.2 (172.2–194.3)	**21.5 (16.3–26.7)**	**− 6.8 (− 13.5 to − 0.1)**
*Change per exercise session*				**1.28 (0.59–1.97)**	0.18 (− 0.71–1.07)
1RM leg press in kg	(n=711)	(n=613)	(n=307)		
*Total population*	88.3 (82.4–94.2)	107.2 (101.2–113.2)	89.8 (83.5–96.1)	**18.9 (15.9–21.9)**	1.5 (− 2.5–5.5)
*Tumor location*^*b*^					
Gastrointestinal	91.0 (84.9–97.1)	110.6 (104.4–116.8)	88.3 (81.5–95.1)	**19.6 (16.1–23.1)**	− 2.7 (− 7.7–2.4)
Urological	78.3 (63.1–93.5)	96.0 (80.6–111.5)	86.4 (70.5–102.2)	**17.7 (9.8–25.7)**	8.1 (− 0.8–16.9)
Gynecological (p_interaction_=0.010)	69.6 (53.6–85.6)	85.9 (69.7–102.1)	77.0 (60.3–93.7)	**16.3 (8.3–24.4)**	7.4 (− 2.0–16.9)
*Neoadjuvant therapy*^*b*^					
No	93.8 (86.7–100.8)	110.4 (103.2–117.5)	95.7 (88.1–103.2)	**16.6 (12.8–20.4)**	1.9 (− 3.0–6.8)
Yes (p_interaction_=0.019)	78.9 (67.6–90.1)	101.5 (90.1–112.8)	78.9 (66.9–90.8)	**22.6 (17.8–27.4)**	0.0 (− 6.8–6.9)
*Malnutrition risk*^*b*^					
Low	96.4 (89.0–103.8)	113.4 (105.9–120.9)	97.7 (89.8–105.5)	**17.0 (13.3–20.7)**	1.3 (− 3.4–6.0)
High (p_interaction_=0.031)	77.9 (68.0–87.7)	100.6 (90.6–110.6)	79.4 (68.7–90.1)	**22.7 (17.6–27.9)**	1.5 (− 5.9–9.0)
*Exercise sessions*^*b*^					
0–6	79.8 (71.3–88.2)	93.5 (84.9–102.2)	81.8 (72.7–91.0)	**13.8 (9.4–18.2)**	2.1 (− 3.7–7.8)
>6 (p_interaction_≤0.001)	96.2 (88.0–104.5)	119.2 (111.0–127.5)	96.8 (88.1–105.6)	**23.0 (19.0–27.0)**	0.6 (− 4.9–6.0)
*Change per exercise session*				**1.00 (0.44–1.55)**	0.31 (− 0.43–1.04)
HGS in kg	(n=803)	(n=699)	(n=365)		
*Total population*	33.4 (32.5–34.4)	34.5 (33.6–35.5)	32.4 (31.4–33.4)	**1.1 (0.6–1.6)**	− **1.1 (− 1.7 to − 0.4)**
*Tumor location*^*b*^					
Gastrointestinal	34.0 (33.1–35.0)	35.4 (34.4–36.4)	32.6 (31.5–33.6)	**1.4 (0.8–1.9)**	**− 1.4 (− 2.2 to − 0.7)**
Urological	33.1 (30;8–35.5)	32.9 (30.5–35.3)	31.5 (29.1–34.0)	− 0.2 (− 1.5–1.1)	**− 1.6 (− 3.1 to − 0.2)**
Gynecological (p_interaction_=0.005)	27.5 (25.1–29.9)	28.6 (26.1–31.1)	28.5 (25.9–31.0)	1.1 (− 0.1–2.3)	1.0 (− 0.5–2.4)
*Neoadjuvant therapy*^*b*^					
No	33.7 (32.6–34.8)	34.5 (33.4–35.6)	32.4 (31.2–33.5)	**0.8 (0.2–1.4)**	**− 1.3 (− 2.1 to − 0.6)**
Yes (p_interaction_=0.025)	32.8 (31.0–34.7)	34.5 (32.6–36.4)	32.3 (30.4–34.2)	**1.7 (1.0–2.4)**	− 0.5 (− 1.5–0.5)
FTSST in seconds	(n=784)	(n=670)	(n=355)		
*Total population*	10.5 (10.0–11.0)	9.6 (9.2–10.1)	10.2 (9.7–10.7)	**− 0.9 (− 1.1 to − 0.6)**	− 0.3 (− 0.6–0.0)
*Tumor location*^*b*^					
Gastrointestinal	10.0 (9.6–10.5)	9.2 (8.8–9.7)	10.0 (9.5–10.5)	**− 0.8 (− 1.1 to − 0.5)**	0.0 (− 0.5–0.4)
Urological	11.9 (10.3–13.4)	10.8 (9.2–12.4)	11.2 (9.6–12.8)	**− 1.1 (− 1.7 to − 0.5)**	− **0.7 (− 1.3 to − 0.1)**
Gynecological (p_interaction_=0.033)	9.8 (8.2–11.4)	8.8 (7.2–10.4)	8.9 (7.3–10.6)	**− 0.9 (− 1.7 to − 0.2)**	− 0.9 (− 1.8–0.0)
*Malnutrition risk*^*b*^					
Low	10.0 (9.5–10.6)	9.4 (8.8–9.9)	9.8 (9.2–10.5)	**− 0.7 (− 1.0 to − 0.4)**	− 0.2 (− 0.6–0.2)
High (p_interaction_=0.044)	11.1 (10.3–11.9)	9.9 (9.1–10.7)	10.6 (9.7–11.4)	**− 1.2 (− 1.7 to − 0.7)**	− 0.5 (− 1.2–0.2)
*Exercise sessions*					
0–6	10.8 (10.1–11.4)	10.0 (9.3–10.7)	10.2 (9.5–10.9)	**− 0.8 (− 1.2 to − 0.4)**	**− 0.6 (− 1.1 to − 0.1)**
>6 (p_interaction_=0.044)	10.1 (9.4–10.8)	9.2 (8.5–9.9)	10.1 (9.4–10.8)	**− 0.9 (− 1.3 to − 0.6)**	0.0 (− 0.5–0.4)

Bold values indicate statistical significance (95% confidence interval does not include 0)

Data are presented as mean (95% confidence interval) unless otherwise indicated.

^a^Models corrected for age, sex, smoking status (no vs. yes), ASA score (I–II vs. III), tumor location (gastrointestinal, gynecological, urological), neoadjuvant treatment (no vs. yes), and malnutrition risk based on Patient-Generated Subjective Global Assessment Short Form scores (0–3 vs. ≥4) as fixed effects, and patient as a random effect.

^b^Also statistically significant when adjusted for the number of exercise sessions.

ASA, American Society of Anesthesiologists; FTSST, five-times sit-to-stand; HGS, handgrip strength; MSEC, maximal short-time exercise capacity; peakVO_2_, maximal oxygen uptake; 1RM, 1 repetition maximum.

Overall, the MSEC increased significantly from baseline to postintervention (*β*=17.5 Watt, 95% CI 13.8–21.2). Larger increases were found in patients with abdominal tumor locations (*p*_interaction_ = 0.020), those receiving neoadjuvant treatment (*p*_interaction_ = <0.001), and when attending more than six exercise sessions (*p*_interaction_ = 0.004). Notably, these same groups experienced more pronounced post-surgical declines, returning to baseline levels or, in some cases, falling significantly below baseline levels. There was a positive exercise dose–response association (per exercise session: *β*=1.28, 95% CI 0.59–1.97).

1RM leg press significantly increased from baseline to postintervention (*β* = 18.9 kg 95% CI 15.9–21.9), with significantly larger increases among patients undergoing neoadjuvant therapy (*p*_interaction_ = 0.019), at risk of malnutrition (*p*_interaction_ = 0.031), or highly adherent to the exercise program (*p*_interaction_ = < 0.001). Also, dose–response associations were found (per exercise session: *β*=1.00 kg, 95% CI 0.44–1.55). Post-surgical values regressed to baseline.

HGS demonstrated a significant increase between baseline and postintervention (*β*=1.1 kg, 95% CI 0.6–1.6). The greatest increases were observed in patients with gastrointestinal tumors (*p*_interaction_ = 0.005) and those who had received neoadjuvant treatment (*p*_interaction_ = 0.025). No significant dose–response association was found. At post-surgical follow-up, HGS fell below baseline levels (*β*=1.1 kg, 95% CI  − 1.7 to  − 0.4), with patients with urological tumors and those who had not received neoadjuvant treatment demonstrating the largest decreases.

Lastly, FTSST performance increased during prehabilitation (*β*= − 0.9 seconds, 95% CI  − 1.1 to  − 0.6), with the most notable increases seen in patients at high risk of malnutrition (*p*_interaction_ = 0.044). However, no significant dose–response association was observed for FTSST. Post-surgical FTSST measurements returned to baseline levels.

### Changes in body composition over time, modifying factors, and dose–response associations

During the prehabilitation period, body weight (*β*=1.1 kg, 95% CI 0.7–1.4) and FFM (*β*=0.5 kg, 95% CI 0.1–0.8) increased significantly (Table 3). Larger increases in FFM were observed in patients with gastrointestinal tumors (*p*_interaction_ = 0.025), those who had undergone neoadjuvant therapy (*p*_interaction_ <0.001), individuals at high risk of malnutrition (*p*_interaction_ = 0.047), and those who participated in more than six exercise sessions (*p*_interaction_ <0.001). An exercise dose–response relationship was found for both body weight (per exercise session: *β*=0.11 kg, 95% CI 0.05–0.17) and FFM (per exercise session: *β*=0.12 kg, 95% CI 0.05–0.19). At post-surgical follow-up, body weight (*β*= − 1.7 kg, 95% CI  − 2.1 to  − 1.3) and FFM (*β*= − 1.1 kg, 95% CI  − 1.6 to  − 0.6) declined significantly below baseline values. Post-surgical weight loss was most pronounced in patients with gastrointestinal tumors and those who received neoadjuvant therapy. The greatest postoperative FFM loss occurred in patients with gastrointestinal tumors, who received neoadjuvant therapy, who had a lower risk of malnutrition, or who attended six or fewer exercise sessions.

**Table 3:** Descriptives of body composition parameters at all timepoints and changes over time with interaction terms

Body composition parameters	Preintervention	Postintervention	Post-surgical follow-up (3 months)	Difference between baseline to postintervention, *β* (95% CI)^a^	Difference between baseline and post-surgical follow-up, *β* (95% CI)^a^
*Body composition*					
Body weight, kg	(*n*=805)	(*n*=705)	(*n*=368)		
*Total population*	79.7 (77.8–81.6)	80.8 (78.9–82.7)	78.0 (76.1–79.9)	**1.1 (0.7–1.4)**	**− 1.7 (− 2.1 to − 1.3)**
*Tumor location*^*b*^					
Gastrointestinal	76.8 (74.9–78.7)	78.1 (76.2–80.1)	74.1 (72.1–76.0)	**1.3 (0.9–1.7)**	**− 2.7 (− 3.3 to − 2.2)**
Urological	80.4 (75.6–85.2)	80.7 (75.9–85.5)	79.7 (74.9–84.6)	0.3 (− 0.4–1.0)	− 0.7 (− 1.4–0.1)
Gynecological (p_interaction_=<.001)	73.8 (68.6–78.9)	74.2 (69.0–79.4)	74.7 (69.5–79.9)	0.4 (− 0.1–1.0)	**0.9 (0.2;1.6)**
*Neoadjuvant therapy*^*b*^					
No	81.5 (79.1–83.9)	82.0 (79.6–84.4)	80.2 (77.8–82.6)	**0.6 (0.2–0.9)**	**− 1.3 (− 1.8 to − 0.8)**
Yes (p_interaction_=<.001)	77.6 (74.4–80.9)	79.6 (76.3–82.8)	75.0 (71.7–78.2)	**1.9 (1.4–2.5)**	**− 2.7 (− 3.4 to − 1.9)**
*Malnutrition risk*^*b*^					
Low	81.3 (79.1–83.5)	82.0 (79.8–84.3)	79.7 (77.4–81.9)	**0.8 (0.4–1.1)**	**− 1.6 (− 2.1 to − 1.2)**
High (p_interaction_<.001)	78.3 (74.9–81.8)	80.0 (76.6–83.5)	76.4 (72.9–79.9)	**1.7 (1.1–2.3)**	**− 1.9 (− 2.8 to − 1.1)**
*Exercise sessions*					
0–6	78.6 (76.0–81.1)	79.2 (76.6–81.7)	76.8 (74.3–79.4)	**0.6 (0.2–1.0)**	**− 1.7 (− 2.3 to − 1.2)**
>6 (p_interaction_=0.002)	80.5 (77.5–83.4)	81.9 (79.0–84.8)	78.8 (75.9–81.7)	**1.4 (1.0–1.9)**	**− 1.7 (− 2.3 to − 1.1)**
*Change per exercise session*				**0.11 (0.05–0.17)**	0.00 (− 0.07–0.08)
FFM, kg	(n=775)	(n=659)	(n=353)		
*Total population*	53.5 (52.5–54.4)	53.9 (52.9–54.9)	52.3 (51.3–53.3)	**0.5 (0.1–0.8)**	**− 1.1 (− 1.6 to − 0.6)**
*Tumor location*^*b*^					
Gastrointestinal	52.2 (51.2–53.2)	52.9 (51.9–53.9)	50.6 (49.5–51.6)	**0.7 (0.3–1.1)**	**− 1.6 (− 2.2 to − 1.0)**
Urological	54.4 (51.1–57.0)	54.1 (51.4–56.8)	53.7 (51.0–56.4)	− 0.3 (− 1.2–0.6)	− 0.7 (− 1.7–0.3)
Gynecological (p_interaction_=0.025)	45.7 (43.4–48.1)	45.7 (43.3–48.1)	45.9 (43.5–48.4)	0.0 (− 1.0–1.0)	0.2 (− 1.0–1.3)
*Neoadjuvant therapy*^*b*^					
No	53.8 (52.6–55.0)	53.9 (52.7–55.1)	52.8 (51.6–54.1)	0.0 (− 0.4–0.5)	**− 1.0 (− 1.6 to − 0.4)**
Yes (p_interaction_<.001)	53.4 (51.7–55.2)	54.6 (52.9–56.4)	52.0 (50.3–53.8)	**1.2 (0.6–1.8)**	**− 1.4 (− 2.2 to − 0.6)**
*Malnutrition risk*^*b*^					
Low	54.1 (53.0–55.3)	54.4 (53.2–55.5)	52.8 (51.6–54.0)	0.3 (− 0.2–0.7)	**− 1.3 (− 1.9 to − 0.8)**
High (p_interaction_=0.047)	52.8 (51.0–54.5)	53.7 (51.9–55.4)	52.1 (50.3–53.9)	**0.9 (0.3–1.5)**	− 0.7 (− 1.5–0.2)
*Exercise sessions*					
0–6	52.9 (51.6–54.1)	52.8 (51.5–54.1)	51.4 (50.0–52.7)	− 0.1 (− 0.6–0.5)	**− 1.5 (− 2.2 to − 0.8)**
>6 (p_interaction_<.001)	53.8 (52.3–55.3)	54.7 (53.2–56.2)	53.0 (51.5–54.5)	**0.9 (0.4–1.4)**	**− 0.8 (− 1.4 to − 0.1)**
*Change per exercise session*				**0.12 (0.05–0.19)**	0.08 (0.00–0.17)
Fat percentage^c^	(n=775)	(n=659)	(n=353)		
*Total population*	32.6 (31.8–33.3)	33.0 (32.2–33.7)	32.5 (31.7–33.3)	0.4 (− 0.0–0.8)	− 0.1 (− 0.6–0.5)
Phase angle in degrees^c^	(n=775)	(n=659)	(n=353)		
*Total population*	5.4 (5.1–5.8)	5.7 (5.3–6.1)	5.5 (5.0–5.9)	0.3 (− 0.2–0.8)	0.0 (− 0.5–0.6)

Bold values indicate statistical significance (95% confidence interval does not include 0)

Data are presented as mean (95% confidence interval) unless otherwise indicated

FFM, fat-free mass

^a^Models corrected for age, sex, smoking status (no vs. yes), American Society of Anesthesiologists score (I–II vs. III), tumor location (gastrointestinal, gynecological, urological), neoadjuvant treatment (no vs. yes), and malnutrition risk based on Patient-Generated Subjective Global Assessment Short Form scores (0–3 vs. ≥4) as fixed effects, and patient as a random effect

^b^Also statistically significant when adjusted for the number of exercise sessions

^c^For these body composition outcomes none of the interaction terms reached statistical significance

In contrast, both fat percentage and phase angle remained stable, with no significant changes observed either after prehabilitation or at post-surgical follow-up. Moreover, no dose–response associations were identified for either parameter.

### Sensitivity Analyses

The sensitivity analysis among 257 patients with complete data at both the preintervention and the post-surgical follow-up measurement showed that the effect estimates were somewhat attenuated (Supplementary Table 1), but the findings after subgroup analysis were consistent with the results of the total group.

## Discussion

This study evaluated the preoperative to postoperative changes in functional capacity and body composition in patients undergoing abdominal surgery for cancer following a multimodal prehabilitation program. We observed increases in all key measures of functional capacity, body weight, and FFM, although many of these gains regressed postoperatively, often returning to or falling below baseline levels. The course of functional capacity and body composition varied by tumor location, neoadjuvant therapy, risk of malnutrition, and compliance with exercise sessions. Notably, we found significant exercise dose–response associations for aerobic fitness, muscle strength, body weight, and FFM.

The observed preoperative increases in functional capacity support previous findings that multimodal prehabilitation can enhance fitness before surgery, most often measured by the 6-minute walk distance.^14,29^ In contrast, several trials reported no improvement in VO_2_ peak compared with the control group.^14,30^ These discrepancies may reflect differences in baseline patient characteristics, suggesting that responses to prehabilitation are heterogeneous and may depend on patient selection. However, in line with two smaller randomized controlled trials, our study also demonstrated that functional capacity frequently declined after surgery, returning to baseline levels.^31,32^ Whereas most previous studies assessed functional capacity using the 6-minute walk test, our study employed multiple objective measures, providing a broader evaluation of functional status. In addition, our longitudinal design allowed for a more detailed assessment of the recovery trajectory over time. This revealed that – even with prehabilitation – functional declines after surgery are common, underscoring the lasting physiological impact of major surgery, which affects patients’ quality of recovery.^33^ These findings suggest that prehabilitation, as a stand-alone intervention, may be suboptimal to achieve timely functional recovery post-surgery, emphasizing the potential need for subsequent postoperative rehabilitation. Recent evidence indicates that structured postoperative exercise programs are feasible, can improve physical performance, and can enhance treatment tolerability in patients with cancer undergoing abdominal surgery.^34,35^ Future trials are warranted to evaluate the combined effect of prehabilitation and postoperative rehabilitation on functional recovery.

Beyond functional measures, this study also demonstrated increases in body weight and FFM during the preoperative period. These findings are consistent with earlier research suggesting that prehabilitation may preserve skeletal muscle during neoadjuvant therapy and can promote increases in muscle mass after the intervention.^36,37^ Our focus on both pre- and postoperative body composition trajectories in the context of multimodal prehabilitation confirms that muscle mass can be improved preoperatively but that these gains are lost after surgery. Given that muscle loss has been strongly associated with delayed recovery and reduced functional independence, these findings emphasize the importance of regaining muscle mass after surgery.^18,38^ This could be achieved by progressive resistance exercise training and adequate protein and energy intake after surgery.^39,40^

Examinations of dose–response associations addressed an important research gap. Larger increases in functional capacity and body composition may enhance resilience to surgical stress and reduce postoperative complications, including pulmonary events and overall morbidity.^41^ Although we found stronger associations among patients who completed more than the median of six exercise sessions, future studies should focus on the minimum number of exercise sessions associated with meaningful changes in functional capacity and body composition outcomes. At the same time, we also observed significant increases in functional capacity in patients who completed fewer than six exercise sessions. This has practical implications for patients with limited time before surgery and for those less inclined to engage in prehabilitation programs. In contrast, those who completed six or fewer exercise sessions did not increase FFM preoperatively and experienced the greatest losses after surgery. These findings support the potential value of personalized exercise dosing, in which patients at higher risk may benefit from more extended training programs to optimize functional recovery.

The identification of patient populations showing larger increases remains another important research gap.^16^ We found that patients who had received neoadjuvant therapy or were at risk of malnutrition experienced the largest gains in both functional capacity and FFM. This may be explained by lower baseline fitness levels, allowing greater potential for improvement.^42^ These findings underscore the relevance of patient-specific risk profiling to target prehabilitation to patients most in need and demonstrate that even clinically vulnerable populations can respond positively to structured prehabilitation interventions. However, patients with an ASA score of IV were excluded for safety reasons, which limits the generalizability of our findings. At the same time, it remains unclear whether the observed increases in functional capacity and body composition are clinically meaningful or translate to improved postoperative outcomes. Future research is needed to determine the minimal gain in functional capacity and body composition required to improve postoperative outcomes. In addition, recovery trajectories vary substantially across different surgical procedures. Better characterization of the timing and extent of recovery in specific surgical populations is needed to enable more targeted and timely postoperative rehabilitation strategies.

A strength of this study is the large and heterogeneous sample of patients with abdominal cancer undergoing multimodal prehabilitation. This large sample size enabled investigations of clinical variables modifying the changes over time and the dose–response associations. Moreover, the inclusion of diverse tumor types and surgical procedures enhanced generalizability. However, a limitation of this study is the absence of a control group, which restricts our ability to draw conclusions about causal effects of prehabilitation. Patient engagement with assessments in the absence of an intervention was low, and the stepped wedge design led to a rapid transition of most care pathways from control to intervention, limiting the size of the control cohort. Additionally, there was a substantial proportion of missing data, particularly at the postoperative time point. Although the main reasons were not systematically recorded, they were practical constraints on the day of surgery (e.g., fasting requirements prohibiting exercise testing), loss to follow-up, and patient enrollment before the postsurgical measurement was added to the protocol. Although linear mixed models account for data missing at random, we acknowledge that estimates can be sensitive to the high level of missingness. Our sensitivity analysis in the subgroup of patients with complete data at both the preintervention and post-surgical follow-up time point did not change our conclusion. Notably, the somewhat attenuated changes in this subgroup may be explained by the better baseline values, leaving less room for improvement. Additional analyses also indicated that active smokers and patients with an ASA score of III were more likely to have missing data at follow-up measurements, which should be considered when interpreting our findings. Furthermore, a limitation of our study is the limited reporting of adverse events during the exercise intervention, as documentation relied on physical therapists and may have resulted in underreporting, whereas the completion of specific exercise components or any modifications made during sessions were not systematically documented, highlighting the need for closer monitoring in future studies.

## Conclusions

This study demonstrated that patients undergoing abdominal surgery for cancer after participating in a multimodal prehabilitation program showed preoperative increases in functional capacity and body composition. However, postoperative declines were common and often resulted in a return to or deterioration below baseline levels. These trajectories were influenced by tumor location, neoadjuvant therapy, malnutrition risk, and compliance with exercise sessions. Importantly, we found significant exercise dose–response associations for estimated peakVO_2_, MSEC, 1RM, body weight, and FFM. These findings underscore the substantial physiological impact of surgery and suggest that patient-specific approaches to prehabilitation, as well as integration with postoperative rehabilitation and nutritional support, warrant further investigation.

## Supplementary Information

Below is the link to the electronic supplementary material.

Supplementary file1 (PDF 208 KB)

Supplementary file1 (DOCX 18 KB)

## Data Availability

The datasets used and/or analyzed during the current study are available from the corresponding author on reasonable request.
