# Supplementary material for: Changes in Functional Capacity and Body Composition After a Multimodal Prehabilitation Program in Patients with Cancer undergoing Abdominal Surgery
Source: Ann Surg Oncol. 2026 Feb 23;33(6):5714–25. doi: 10.1245/s10434-026-19173-4 (PMC13179190; doi:10.1245/s10434-026-19173-4)
Supplement: Supplementary file 1 — Supplementary file1 (PDF 208 KB) [file 10434_2026_19173_MOESM1_ESM.pdf]

Data-analysis plan Functional Capacity and Body Composition after Multimodal Prehabilitation in Patients with Cancer undergoing Abdominal Surgery

Working title

The Effects of a Multimodal Prehabilitation Program on Preoperative and Postoperative Functional Capacity and Nutritional Status in Patients undergoing Major Abdominal Cancer Surgery

Aims of the Study

Overall aim:

To investigate the longitudinal, confounder-adjusted changes in functional capacity and body composition during and after multimodal prehabilitation, and to evaluate exercise dose-response associations in patients undergoing elective abdominal cancer surgery. Data were derived from three repeated assessments (baseline, post-intervention, and three months postoperatively) within the F4S stepped-wedge trial.

Primary aim:

To assess longitudinal changes in functional capacity and body composition outcomes over time.

Secondary aim:

To evaluate dose-response associations between exercise session compliance and these outcomes.

Timeline

- Data analysis plan – October 2024 – November 2024
- Analyses – January 2025 – April 2025
- Writing of the manuscript and first draft – April 2025 – October 2025

Introduction

Patients undergoing major abdominal surgery experience a significant amount of postoperative complications (1). In cases involving malignancy, preoperative physical deterioration is common, driven by disease-related symptoms and the adverse effects of neoadjuvant treatment (2). Since preoperative functional capacity is closely associated with postoperative outcomes, efforts to minimize functional decline during the preoperative period are essential (3).

Multimodal prehabilitation, which integrates physical exercise, nutritional support, psychological assistance, and a smoking cessation intervention, has emerged as a promising approach to enhance preoperative physiological reserves in surgical patients (4, 5). For individuals undergoing colorectal cancer surgery, multimodal prehabilitation has demonstrated beneficial effects, such as a reduction in postoperative complications and enhanced postoperative recovery (6).

However, despite the growing evidence supporting multimodal prehabilitation, there is limited evidence on the progression of body composition and functional capacity throughout the preoperative and postoperative periods. Additionally, there is uncertainty regarding the optimal duration of prehabilitation required to maximize benefits, and the extent to which a single exercise session influences these parameters has not been well established. In order to optimize prehabilitation protocols and improving patient outcomes, understanding these factors is critical.

This study will assess the changes after a multimodal prehabilitation program on preoperative and postoperative body composition and functional capacity in a large cohort of patients undergoing major abdominal cancer surgery. Additionally, it will evaluate the associations between the number of exercise sessions and these outcomes.

Study population

In the present study data is derived from the F4S PREHAB trial, a monocenter stepped wedge trial designed to evaluate the hospital-wide effects of a multimodal prehabilitation program in patients undergoing various high-impact surgical procedures (7). Ethical approval for the F4S PREHAB trial was granted by the local Medical Ethics Committee (METC Oost-Nederland; NL73777.091.20).

For the present study, patients were included if they underwent elective surgery for various types of abdominal cancer from 1 March 2021 up until 30 April 2024 in the Radboudumc (Nijmegen, the Netherlands). Abdominal cancers included gastro-intestinal cancers (colon cancer, rectal cancer, liver cancer or colorectal liver metastases, peritoneal metastases, retroperitoneal sarcomas, esophageal cancer, and pancreatic cancer), gynecological cancers (endometrial and ovarian cancer), and urological cancers (bladder and renal cancer). Patients were excluded from participating in the F4S PREHAB trial if they had contraindications for high-intensity exercise or protein supplementation, an American Society of Anesthesiologists (ASA) score ≥ 4, or inability to read or understand the Dutch language. To investigate changes in functional capacity and body composition in the post-operative period, we added these measurements after the start of the trial (August 2022).

Main outcomes

Primary outcomes – Changes in pre-post intervention and postoperatively (+3 months)

- Body composition / nutritional parameters
    - Fat-free mass in kg (Bioelectrical Impedance Analysis)
    - Fat percentage (Bioelectrical Impedance Analysis)
    - Phase angle in degrees (Bioelectrical Impedance Analysis)
- Physical functioning parameters
    - Aerobic fitness (estimated VO_2 Max in mL/kg/min – Steep Ramp Test)
    - MSEC in watt (Steep Ramp Test)
    - Leg muscle strength in kg (one repetition maximum – Leg press)
    - Hand grip strength in kg (hand grip strength)
    - Lower limb functioning (Five Times Sit to Stand Test in seconds)

Statistical analyses

<u>Changes in pre-post intervention and postoperatively (+3 months)</u>

Changes from pre- to post-intervention and postoperative follow-up will be assessed using linear mixed models. This model handles missing data under the missing-at-random assumption.
We assume that the data missing at T2 measurement (because this was added after the start of the trial) will be missing at random. To check whether missing data are selective, we will use logistic regression analyses to examine differences in baseline characteristics between patients with complete data and patients with missing values.

Linear mixed models

- Dependent variables (outcome):
    - Weight in kg
    - Fat-free mass in kg (Bioelectrical Impedance Analysis)
    - Fat percentage (Bioelectrical Impedance Analysis)
    - Phase angle (Bioelectrical Impedance Analysis)
    - Aerobic fitness (estimated VO_2 Max in mL/kg/min – Steep Ramp Test)
    - MSEC in watt (Steep Ramp Test)
    - Leg muscle strength in kg (one repetition maximum – Leg press)
    - Hand grip strength in kg (hand grip strength)
    - Lower limb functioning (Five Times Sit to Stand Test in seconds)

- Fixed variables:
    - Age in years
    - Sex (male vs. female)
    - Smoking status (no vs. yes)
    - ASA score (I-II vs. III)
    - Tumor location (abdominal vs. gynecological vs. urological)
    - Neoadjuvant treatment (yes vs. no)
    - Malnutrition risk (0-3 vs. ≥4)

- Random intercepts:
    - Participants

Additionally, interaction terms will be added to the models to evaluate potential differences in changes over time between subgroups.

- Interaction terms:
    - Visit * tumor location
    - Visit * neoadjuvant treatment
    - Visit * malnutrition risk

Relationship between the number of exercise sessions and changes in outcomes (dose-response)

Linear mixed models

- Dependent variables (outcome):
  - Weight in kg
  - Fat-free mass in kg (Bioelectrical Impedance Analysis)
  - Fat percentage (Bioelectrical Impedance Analysis)
  - Phase angle (Bioelectrical Impedance Analysis)
  - Aerobic fitness (estimated VO_2 Max in mL/kg/min – Steep Ramp Test)
  - MSEC in watt (Steep Ramp Test)
  - Leg muscle strength in kg (one repetition maximum – Leg press)
  - Hand grip strength in kg (hand grip strength)
  - Lower limb functioning (Five Times Sit to Stand Test in seconds)

- Fixed variables:
  - Age in years
  - Sex (male vs. female)
  - Smoking status (no vs. yes)
  - ASA score (I-II vs. III)
  - Tumour location (abdominal vs. gynecological vs. urological)
  - Neoadjuvant treatment (yes vs. no)
  - Malnutrition risk (0-3 vs. ≥4)
  - Exercise sessions

- Random intercepts:
  - Participants

- Interaction terms:
    - Visit * exercise sessions

Draft list of authors

L.D. Drager, L.M. Buffart, D. Strijker, S. Verlaan, C.J.H.M. van Laarhoven, B. van den Heuvel

References

1. Helden EV, Kranendonk J, Vermulst A, Boer A, Reuver P, Rosman C, et al. Early postoperative pain and 30-day complications following major abdominal surgery: a retrospective cohort study. Reg Anesth Pain Med. 2024.
2. Guinan EM, Doyle SL, Bennett AE, O'Neill L, Gannon J, Elliott JA, et al. Sarcopenia during neoadjuvant therapy for oesophageal cancer: characterising the impact on muscle strength and physical performance. Support Care Cancer. 2018;26(5):1569-76.
3. Heldens AFJM, Bongers BC, Lenssen AF, Stassen LPS, Buhre WF, van Meeteren NLU. The association between performance parameters of physical fitness and postoperative outcomes in patients undergoing colorectal surgery: An evaluation of care data. Ejso-Eur J Surg Onc. 2017;43(11):2084-92.
4. Chen BP, Awasthi R, Sweet SN, Minnella EM, Bergdahl A, Santa Mina D, et al. Four-week prehabilitation program is sufficient to modify exercise behaviors and improve preoperative functional walking capacity in patients with colorectal cancer. Support Care Cancer. 2017;25(1):33-40.
5. Barberan-Garcia A, Ubre M, Roca J, Lacy AM, Burgos F, Risco R, et al. Personalised Prehabilitation in High-risk Patients Undergoing Elective Major Abdominal Surgery: A Randomized Blinded Controlled Trial. Ann Surg. 2018;267(1):50-6.
6. Molenaar CJL, Minnella EM, Coca-Martinez M, Ten Cate DWG, Regis M, Awasthi R, et al. Effect of Multimodal Prehabilitation on Reducing Postoperative Complications and Enhancing Functional Capacity Following Colorectal Cancer Surgery: The PREHAB Randomized Clinical Trial. JAMA Surg. 2023;158(6):572-81.
7. Strijker D, Drager L, van Asseldonk M, Atsma F, van den Berg M, van Daal E, et al. Multimodal prehabilitation (Fit4Surgery) in high-impact surgery to enhance surgical outcomes: Study protocol of F4S PREHAB, a single center stepped wedge trial. Plos One. 2024;19(7).
