# Supplementary material for: Changes in Functional Capacity and Body Composition After a Multimodal Prehabilitation Program in Patients with Cancer undergoing Abdominal Surgery
Source: Ann Surg Oncol. 2026 Feb 23;33(6):5714–25. doi: 10.1245/s10434-026-19173-4 (PMC13179190; doi:10.1245/s10434-026-19173-4)
Supplement: Supplementary file 2 — Supplementary file1 (DOCX 18 KB) [file 10434_2026_19173_MOESM2_ESM.docx]

**Supplementary Table 1. Descriptives of functional capacity parameters at all timepoints and changes over time with interaction terms in the 257 patients with complete T0 and T2 data**

|  | **Pre-intervention,** mean (95% CI) | **Post-intervention,** mean (95% CI) | **Post-surgical follow-up (3 months),** mean (95% CI) | **Difference between baseline to post-intervention,**  β (95% CI)* | **Difference between baseline and post-surgical follow-up,**  β (95% CI)* |
| --- | --- | --- | --- | --- | --- |
| **Functional capacity** | | | | | |
| **Estimated peakVO_2_ in mL/kg/min** | 21.2 (20.3; 22.1) | 21.7 (20.8; 22.6) | 21.2 (20.3; 22.1) | **0.5 (0.2; 0.8)** | 0.0 (-0.3; 0.2) |
| **MSEC in Watt** | 201.8 (188.4; 215.2) | 216.5 (203.0; 230.0) | 194.1 (180.7; 207.5) | **14.7 (8.6; 20.9)** | **-7.7 (-13.6; -1.8)** |
| **1RM leg press in kg** | 106.4 (95.7; 117.1) | 121.6 (110.8; 132.4) | 106.5 (95.8; 117.2) | **15.2 (10.3; 20.0)** | 0.0 (-4.6; 4.7) |
| **HGS in kg** | 34.3 (32.5; 36.0) | 35.1 (33.4; 36.8) | 32.9 (31.2; 34.6) | **0.8 (0.0; 1.6)** | **-1.3 (-2.1; -0.6)** |
| **FTSST in seconds** | 9.2 (8.5; 9.8) | 8.3 (7.7; 9.0) | 8.9 (8.3; 9.6) | **-0.8 (-1.2; -0.5)** | -0.2 (-0.6; -0.1) |
| **Body composition** | | | | | |
| **Body weight in kg** | 81.6 (78.2; 85.0) | 82.6 (79.2; 86.0) | 80.1 (76.7; 83.5) | **1.0 (0.4; 1.6)** | **-1.5 (-2.1; -1.0)** |
| **FFM in kg** | 55.1 (53.3; 56.8) | 55.1 (53.3; 56.8) | 53.8 (52.0; 55.5) | 0.0 (-0.6; 0.7) | **-1.3 (-1.9; -0.7)** |
| **Fat percentage** | 32.2 (30.8; 33.7) | 33.0 (31.6; 34.5) | 32.4 (30.9; 33.8) | **0.8 (0.1; 1.5)** | 0.2 (-0.5; 0.9) |
| **Phase angle in degrees** | 5.4 (4.8; 6.0) | 5.8 (5.2; 6.4) | 5.3 (4.7; 5.9) | 0.4 (-0.3; 1.1) | -0.1 (-0.7; 0.6) |
| * Models corrected for: age, sex, smoking status (no vs. yes), ASA score (1-2 vs. 3), tumor location (gastrointestinal, gynecological, urological), neoadjuvant treatment (no vs. yes), and malnutrition risk based on PG-SGA SF scores (0-3 vs. ≥4) as fixed effects, and patient as a random effect  **Abbreviations**: peakVO_2_; maximal oxygen uptake, MSEC; maximal short-time exercise capacity, 1RM; 1 repetition maximum, HGS; handgrip strength, FTSST; five-times sit-to-stand | | | | | |
